# Assessing the accuracy of predictive models with interval-censored data

**DOI:** 10.1093/biostatistics/kxaa011

**Published:** 2020-03-14

**Authors:** Ying Wu, Richard J Cook

**Affiliations:** 1 School of Statistics and Data Science, Nankai University, Tianjin, 300071, China; 2 Department of Statistics and Actuarial Science, University of Waterloo, Waterloo, ON N2L 3G1, Canada

**Keywords:** Augmented inverse probability weighted estimator, Intermittent assessment, Interval censoring, Inverse probability weighted estimator, Prediction error, ROC curve

## Abstract

We develop methods for assessing the predictive accuracy of a given event time model when the validation sample is comprised of case $K$ interval-censored data. An imputation-based, an inverse probability weighted (IPW), and an augmented inverse probability weighted (AIPW) estimator are developed and evaluated for the mean prediction error and the area under the receiver operating characteristic curve when the goal is to predict event status at a landmark time. The weights used for the IPW and AIPW estimators are obtained by fitting a multistate model which jointly considers the event process, the recurrent assessment process, and loss to follow-up. We empirically investigate the performance of the proposed methods and illustrate their application in the context of a motivating rheumatology study in which human leukocyte antigen markers are used to predict disease progression status in patients with psoriatic arthritis.

## 1. Introduction

Progress towards personalized medicine will depend critically on the development and evaluation of predictive models (Ginsburg and McCarthy, 2001). Accurate predictive models provide a basis for clinical decision-making which can be informed by the anticipated outcomes patients may experience (Gerds *and others*, 2008). Common approaches for quantifying the overall performance of a prediction model are based on explained variation (Korn and Simon, 1990, 1991), the Brier score (Brier, 1950), and loss functions (Wald, 1950). Loss functions measure the distance between the predicted and observed values and, when averaged over all possible data realizations, yield a measure of the prediction error. Validation in an independent external data set is the best way to assess the performance of a predictive model. In the absence of an independent validation sample, the prediction error is typically estimated using a model-based estimator, an estimator of the apparent loss, or cross-validation (Korn and Simon, 1990; Efron, 2004; Rosthøj and Keiding, 2004).

There has been much research on the development of predictive models for time to event data when the outcomes are subject to right censoring. Here, rather than predicting the actual event time, it is more common to predict event status at a landmark time of interest (Uno *and others*, 2007). Assessing the accuracy of a predictive model is challenging even in this setting when the validation sample is subject to right censoring since individuals who are censored before this landmark time will have an unknown event status. To address this, Korn and Simon (1990) proposed use of a bounded loss function for predicting survival time, while inverse probability of censoring weights (IPW) have been used by many authors to deal with censored outcomes (Graf *and others*, 1999; Hothorn *and others*, 2006; Gerds and Schumacher, 2006; Lawless and Yuan, 2010).

When the goal is to predict event status of each individual in a validation sample at a landmark time $t_0$, the discriminative ability of a predictive model can be represented by a receiver operating characteristic (ROC) curve constructed based on the sensitivity and specificity of the classification procedure. Akritas (1994) proposed an estimator based on a nearest neighbor kernel method for the bivariate distribution function of the predictor and the response subject to right censoring. An alternative simple estimator is based on the Kaplan–Meier estimate (Heagerty *and others*, 2000) and Yuan (2008) discussed an estimator for right-censored data using inverse probability of censoring weights.

We consider the problem of assessing the predictive accuracy of a failure time model for the event status (event-free or not) at a landmark time. We suppose data are obtained from a clinical registry of individuals with a chronic disease who are under intermittent observation. The event is only observable upon examination (e.g., by radiological examination) and so the event status is only determined at clinic visits; this observation scheme leads to case $K$ interval-censored data (Sun, 2006). In a validation sample, the concordance of the predicted and actual event status at the landmark time can only be determined for the subset of individuals whose censoring intervals do not span the landmark time. Imputation-based techniques can be employed but related estimates can be seriously biased when the model is misspecified. Alternatively, one may restrict attention to individuals who can be definitively classified but thus leads to a biased validation sub-sample. We propose a joint model for the event process, assessment process, and loss to follow-up time which enables the computation of weights needed to address the biased validation sub-sample. Augmentation terms are also developed to increase the efficiency of the weighted estimators of the predictive accuracy and explore robustness.

The remainder of this article is organized as follows. In Section 2, we describe the nature of the observation process leading to case $K$ interval-censored data and define notation. We describe imputation-based and IPW estimates of prediction error in Section 3.1, and AIPW estimators of the prediction error in Section 3.2 which use both imputation and weighting. Weights are estimated by introducing a joint multistate model for the event process, assessment process, and loss to follow-up; the construction and assumptions justifying the factorization of the full likelihood are given in Appendix A of the Supplementary material available at *Biostatistics* online. The corresponding estimators for sensitivity, specificity, and the area under the ROC are then described. Methods for assessing the discriminative power of predictive models based on ROC curves and the area under such curves are described in Section 4. The design of empirical studies are given in Section 5 where we report on the finite sample properties of the various estimators and explore robustness to model misspecification for the visit process. An illustrative example is given in Section 6 involving data from a psoriatic arthritis clinic, and we conclude in Section 7 with some general remarks.

## 2. Validation sample data and a joint model

We let $T$ denote the event time and an event time model can be represented by a progressive 2-state stochastic process with states labeled 0 and 1 representing the conditions of being event-free and post-event, respectively. We consider the setting of a chronic disease process in which mortality rates are sufficiently low that they can be ignored in terms of modeling and risk prediction. Let $Z(s) = I(T \leqslant s)$ record the state occupied at time $s$ and $\{Z(s), 0 \leqslant s\}$ be the corresponding stochastic process. We assume a prediction is obtained from a parametric hazard-based model for $T$ with


$$\begin{align*}
\lim_{\Delta t \downarrow 0} \frac{P(Z(t + \Delta t^-)=1 | Z(t^-)=0, \boldsymbol{X})}{\Delta t} = I( t \leqslant T) \, h(t | \boldsymbol{X}),
\end{align*}$$

which we parameterize with $\theta$ and $\boldsymbol{X}$ denotes a $p \times 1$ covariate vector. We consider the development of a prediction model based on a training sample ${\mathcal T}$ comprised of $n_0$ independent individuals yielding data $D_{\mathcal T}$ and let $\hat{\boldsymbol{\theta}} = \hat{\boldsymbol{\theta}}(D_{\mathcal T})$ denote the maximum likelihood estimator. In Appendix A of the Supplementary material available at *Biostatistics* online, we describe the construction of the full likelihood and outline the assumptions necessary to justify the partial likelihood typically used when fitting regression models for case $K$ interval-censored data; see also Cook and Lawless (2018, 2021).

Then, we consider the evaluation of the prediction model using a validation sample ${\mathcal V}$ of $n$ independent individuals subject to case $K$ interval censoring (Sun, 2006), wherein the times of all visits and loss to follow-up are available. Specifically, suppose the intention is to follow an individual up to an administrative censoring time $B$, and let $R~(R < B)$ denote a random loss to follow-up time. Then if $C = \min(R, B)$, we define $C(s) = I(C \leqslant s)$ and let $\{C(s), 0 < s\}$ denote the counting process for the censoring time. Individuals are observed at a baseline assessment at $a_0 = 0$ and then intermittently at follow-up clinic visits at times denoted by $0 < a_1 < \cdots,$ where $A_r$ denotes the random time of the $r$th visit and $a_r$ is its realized value, $r = 1, \ldots$. We let $A(s) = \sum_{r=1}^{\infty} I(a_r \leqslant s)$ be a right-continuous process counting the number of post-baseline assessments over $(0, s]$ and let ${\rm d} A(s)=A(s)-A(s^-) = 1$ if an assessment is made at time $s$ and ${\rm d}A(s)=0$ otherwise. Since visits can only be made for individuals still on study, the visit process terminates at $C$ so we observe ${\rm d} \bar{A}(s) = Y(s){\rm d} A(s)$, where $Y(s) = I(s\leqslant C)$ and $\bar{A}(t) = \int_{0}^t {\rm d} \bar{A}(s)$. When considered jointly we refer to $\{C(s), A(s), 0< s\}$ as the *observation process* and we observe $Z(s)$ only if ${\rm d} \bar{A}(s)=1$.

Assessing the accuracy of a prediction model is challenging when data in the validation sample are incomplete due to intermittent observation and loss to follow-up, and either imputation or weighting may be used to address this. For right-censored data, contributions from individuals that can be used for validation are often weighted based on the censoring distribution (Robins *and others*, 1994; Gerds and Schumacher, 2006). When individuals are examined intermittently and the event time is interval-censored, contributions from individuals who provide definitive validation data must be weighted in a way that addresses the more complex observation process. We consider a multistate framework for joint consideration of the event, assessment, and loss to follow-up (censoring) processes, and discuss inverse probability weighted (IPW) and augmented inverse probability weighted (AIPW) estimation of the prediction error. If $\left\{(C(s), \bar A(s), Z(s)), 0 < s\right\}$ denotes the joint process then the states in the joint state space can be represented by the triple $(k_C, k_A, k_Z),$ where $k_C$ and $k_Z \in \{0,1\}$ indicate the loss to follow-up and event status, and $k_A \in \{0, 1, 2, \ldots\}$ represents the cumulative number of assessments.

**Fig. 1.**
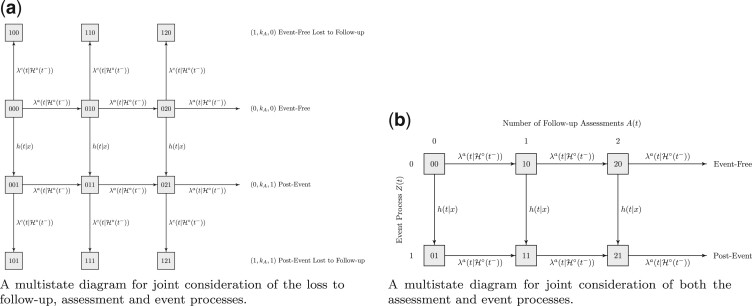
Multistate representation of joint models for observation and event processes. (a) A multistate diagram for joint consideration of the loss to follow-up, assessment, and event processes. (b) A multistate diagram for joint consideration of both the assessment and event processes.

Figure 1(a) displays a state space diagram in which we distinguish between assessments made while an individual is in an event-free state (the second row of states) with those made following event occurrence (the third row of states). Horizontal $(0, k_A, 0) \rightarrow (0, k_A+1, 0)$ transitions correspond to the occurrence of a pre-event assessment, while $(0, k_A, 1) \rightarrow (0, k_A+1, 1)$ transitions correspond to assessments made following event occurrence. To distinguish pre- and post-event assessments notationally, we let ${\rm d} \bar{A}^-(s) = I(s < T) {\rm d} \bar{A}(s)$ and ${\rm d} \bar{A}^+(s) = I(T \leqslant s) {\rm d} \bar{A}(s)$, where ${\rm d} \bar{A}(s) = {\rm d} \bar{A}^-(s) + {\rm d} \bar{A}^+(s)$. The vertical transitions to the top and bottom rows correspond to loss to follow-up pre- and post-event, respectively. For the case $K$ interval-censored data, the parameters of the censoring and assessment processes can be estimated based on the full data likelihood using failure time models for the loss to follow-up time and intensity-based models for the recurrent assessment process using the validation data $D_{\mathcal V} = \{ (a_{ij}, Z(a_{ij}), j = 1, \ldots, m_i, \boldsymbol{X_i}, C_i, \delta_i), i \in {\mathcal V}\}$, where $\delta_i$ is an indication of random drop-out; details are provided in Appendix B of the Supplementary material available at *Biostatistics* online.

In the case when a right censoring time is not recorded, a simplified multistate model can be considered in which the event and intermittent assessment processes are jointly considered as shown in Figure 1(b). The state space is $\{(k_A, k_Z), k_A \in \{0,1,2,\ldots\}, k_Z \in \{0,1\}\}$; a vertical transition reflects the event occurrence and the horizontal transitions to the right reflect the occurrence of the next assessment.

## 3. Prediction error for interval-censored data

### 3.1. Imputation and inverse probability weighting for estimation

Let $t_0$ denote the landmark time at which event status is of interest. A binary status indicator $Y= I(T > t_0)$ indicates that an individual is event-free at $t_0,$ and we let $\hat Y(\boldsymbol{X}; \hat{\boldsymbol\theta})$ denote a prediction for $Y$ based on a model for $Y|\boldsymbol{X}$ indexed by $\boldsymbol\theta$. To examine the accuracy of such a predictive model with a binary response, traditional methods often involve a summary statistic reflecting overall predictive performance such as the mean squared error (Efron, 1983), or a summary statistic for discrimination ability such as the area under a ROC curve (Hanley and McNeil, 1982). We first consider the prediction error based on an absolute error loss function defined as


(3.1) \begin{align*} {\rm PE} = E\left\{ \left| Y - \hat Y(\boldsymbol{X}; \hat{\boldsymbol{\theta}})\right|\right\}, \label{eq:pred_error} \end{align*}

where $\hat{\boldsymbol{\theta}} = \hat{\boldsymbol{\theta}}(D_{\mathcal T})$ is an estimate from a training data set. The optimal predictor is the one that minimizes the prediction error which for (3.1), this is the conditional probability of remaining event-free to $t_0$ given the covariate, $\hat Y(\boldsymbol{X}; \hat{\boldsymbol{\theta}}) = P(T > t_0 | \boldsymbol{X}; \hat{\boldsymbol{\theta}})$. If we focus on the predictor with the same support as $Y,$ then we typically use


(3.2) \begin{align*} \hat Y(\boldsymbol{X}; \hat{\boldsymbol{\theta}}) = I\left(P(T > t_0 | \boldsymbol{X}; \hat{\boldsymbol{\theta}}) > c\right), \end{align*}

which is the optimal binary predictor when the threshold $c = 0.5$ is used; we focus on the binary predictor from here on as this is typically of interest to medical researchers and has been the primary focus for the predictions with right-censored data (Henderson, 1995; Graf *and others*, 1999; Bair and Tibshirani, 2004).

**Fig. 2.**
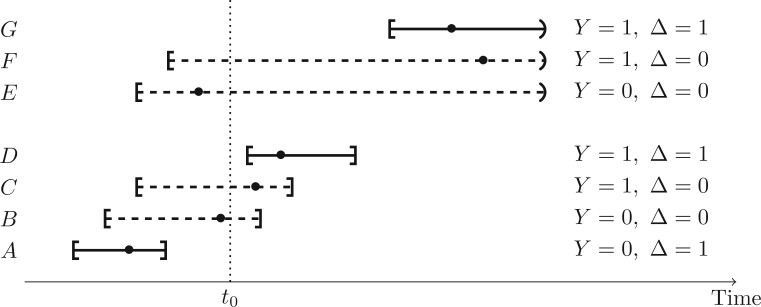
A schematic diagram enumerating possible combinations of $(Y, \Delta)$; the solid lines denote observations in which the event status is known at $t_0$, and the dashed lines denote individual whose event status cannot be classified and hence who are excluded from the sum in (3.3); the solid dots denote the (unobserved) exact event times.

While $\hat Y$ can typically be obtained from a prediction model as discussed in Section 2, the response $Y$ may of course be unknown due to interval censoring. Let $\Delta = I(Y ~\text{is known})$ indicate that the observation status is known. Figure 2 shows all possible combinations of the event status and observation status indicators $(Y, \Delta),$ where the event times for individuals A–D are interval-censored and E–G are right censored. Either imputation or weighting typically are used to deal with the incomplete information in the validation sample. A straightforward imputation-based estimator of the prediction error based on a sample of independent observations of size $n$ is of the form


$$\begin{align*} \widehat{\rm PE}_{\text{IMP}}(t_0) = \frac{1}{n} \sum_{i \in {\mathcal V}} \left[ \Delta_i \left|Y_i - \hat{Y}_i(\boldsymbol{X_i}; \hat{\boldsymbol{\theta}}) \right| + \left( 1 - \Delta_i \right) E \left\{\left|Y_i -\hat{Y}_i(\boldsymbol{X_i}; \hat{\boldsymbol{\theta}})\right| \mid T_i \in [a^-, a^+], \boldsymbol{X_i} \right\} \right], \end{align*}$$

where $a^-$ and $a^+$ are the left and right endpoints of the censoring interval containing the event time. The contributions from individuals with $\Delta_i = 0$ are based on the prediction model, so the performance of this estimator depends on correct specification of the response model and consistent parameter estimation.

To address this, we consider the multistate formulation of the joint observation-event model shown in Figure 1 to construct suitable inverse probability weights when attention is restricted to individuals in a validation sample who can be definitively classified at the landmark time $t_0$. Note that there are two types of individuals with $\Delta=1$. The first type is an individual who is known to have failed before $t_0$ because $\bar{A}^+(t_0) \leqslant 1$; individual A in Figure 2 is an example of such an individual. The second type is an individual known to be event-free after $t_0$ for whom $\bar{A}^+(t_0) = 0$; individuals D and G in Figure 2 are examples of such individuals. Individuals B and C for whom $Y=1$ and 0, respectively have censoring intervals spanning $t_0$ so both have $\Delta =0$, while individuals E and F are right censored before $t_0$ and also have $\Delta=0$.

With a validation sample ${\mathcal V}$ of $n$ independent individuals, and restricting attention to those for whom $\Delta=1$, we can define the IPW estimator of the prediction error as


(3.3) \begin{align*} \widehat{\rm PE}_{\text{IPW}}(t_0)= \frac{1}{n} \sum_{i \in {\mathcal V}} \frac{\Delta_i}{\pi_i} \left|Y_i - \hat Y_i(\boldsymbol{X_i}; \hat{\boldsymbol{\theta}}) \right|, \label{eq:ipw_est} \end{align*}

where the probability $\pi_i$ in the weight is the conditional expectation of $\Delta_i$ given $\left(Y_i, \boldsymbol{X_i}\right)$, or specifically $\pi_i = E(\Delta_i | Y_i, \boldsymbol{X_i})$. Here the distribution of the random variable $\Delta_i$ is governed by the loss to follow-up, assessment, and event processes. The weight can then be written as


(3.4) \begin{align*} \pi_i = E(\Delta_i | Y_i, \boldsymbol{X_i}) = P( \Delta_i = 1 | Y_i, \boldsymbol{X_i}). \end{align*}

This IPW estimate of the prediction error is based on the biased validation sub-sample with $\Delta=1$, but by weighting in (3.3) we obtain a consistent estimator of the prediction error if the joint model is correctly specified. In what follows, we describe how such a joint model can be formed.

We define the intensities for the loss to follow-up and assessment processes as


$$\begin{align*}\lim_{\Delta t \downarrow 0} \frac{P\left(C(t+ \Delta t^-)- C(t^-) = 1 \mid {\mathcal H}(t^-) \right)}{\Delta t}
= I(t \leqslant C) \, \lambda^c(t \mid {\mathcal H}^{\circ}(t^-) )
\end{align*}$$

and


$$\begin{align*}\lim_{\Delta t \downarrow 0} \frac{P\left( \bar{A}(t+ \Delta t^-)- \bar{A}(t^-) = 1 \mid {\mathcal H}(t^{-})\right)}{\Delta t}
= I(t < C) \, \lambda^a(t \mid {\mathcal H}^{\circ}(t^{-})),
\end{align*}$$

respectively, where $\bar{A}(t) = \int_{0}^t {\rm d} I(s\leqslant C) {\rm d} A(s)$ and ${\mathcal H}^{\circ}(t) = \{ C(u), \bar{A}(u), 0 \leqslant u \leqslant t; (a_j, Z(a_j)), j = 1, \ldots, \bar{A}(t), X\}$ is the observed history.

*Expectations when $T \leqslant t_0$ is known: $Y= 0, \Delta=1$*

After the occurrence of the event of interest represented by a $(0, k_A, 0) \rightarrow (0, k_A, 1)$ transition in Figure 1(a), the next event to occur can be a visit corresponding to a $(0, k_A, 1) \rightarrow (1, k_A+ 1, 1)$ transition or loss to follow-up corresponding to a $(0, k_A, 1) \rightarrow (1, k_A, 1)$ transition. For $Y$ to be known in this case, a $(0, k_A, 1) \rightarrow (1, k_A+ 1, 1)$ transition must be made before $t_0$. Thus, $P(\Delta_i = 1| Y_i = 0, \boldsymbol{X_i}) = P( \Delta_i = 1| T_i \leqslant t_0, \boldsymbol{X_i})$ can be written as


(3.5) \begin{align*} \int_0^{t_0} \left[ \int_{t}^{t_0} \lambda^a(u | {\mathcal H}^{\circ}(u^{-})) \exp\left\{ - \int_{t}^u \lambda^a(v | {\mathcal H}^{\circ}(v^{-})) {\rm d}v - \int_{0}^u \lambda^c(v | {\mathcal H}^{\circ}(v^{-})) {\rm d}v \right\} {\rm d}u \right] \times f(t|T_i \leqslant t_0, \boldsymbol{X_i})~{\rm d}t. \label{eq:weight_0} \end{align*}

*Expectations when $T > t_0$ is known : $Y=\Delta=1$*

If $Y$ is known to be one, an event-free assessment is required after $t_0$ in which case $A^+(t_0)=0$. Therefore, a $(0, k_A, 0) \rightarrow (0, k_{A+1}, 0)$ transition must be made after $t_0$, rather than a $(0, k_A, 0) \rightarrow (0, k_A, 1)$ transition for event occurrence, or a $(0, k_A, 0) \rightarrow (1, k_A, 0)$ transition corresponding to loss to follow-up. In this case, $P( \Delta_i = 1| Y_i = 1, \boldsymbol{X_i}) = P( \Delta_i = 1| T_i > t_0, \boldsymbol{X_i})$ is given by


(3.6) \begin{align*} \int_{t_0}^{\infty} \lambda^a(u | {\mathcal H}^{\circ}(u^{-}))\exp\left[- \int_{t_0}^{u}\Bigl\{ \lambda^a(v |{\mathcal H}^{\circ}(v^{-})) + h(v| \boldsymbol{X_i}) \Bigr\} ~ \mathrm{d} v -\int_{0}^{u} \lambda^c(v | {\mathcal H}^{\circ}(v^{-})) ~ \mathrm{d} v \right]~ \mathrm{d} u. \label{eq:weight_1} \end{align*}

Estimates of the observation process intensities $\hat{\lambda}^a(\cdot)$ and $\hat{\lambda}^c(\cdot)$ are required to estimate the weights $\hat{\pi}_i$ based on (3.5) and (3.6) which enables construction of (3.3).

In the simplified setting where there is no loss to follow-up, expressions (3.5) and (3.6) reduce to


(3.7) \begin{align*} \int_0^{t_0} \left[ \int_{t}^{t_0} \lambda^a(u | {\mathcal H}^{\circ}(u^{-})) \exp\left\{ - \int_{t}^u \lambda^a(v | {\mathcal H}^{\circ}(v^{-})) ~ \mathrm{d} v \right\} \mathrm{d} u \right] \times f(t|T_i \leqslant t_0, \boldsymbol{X_i}) ~\mathrm{d} t, \label{eq:weight_0_simple} \end{align*}

and


(3.8) \begin{align*} \int_{t_0}^{B} \lambda^a(u|{\mathcal H}^{\circ}(u^{-})) \exp\left[- \int_{t_0}^{u}\Bigl\{ \lambda^a(v|{\mathcal H}^{\circ}(v^{-})) + h(v|\boldsymbol{X_i}) \Bigr\} ~ \mathrm{d} v \right] ~ \mathrm{d} u, \label{eq:weight_1_simple} \end{align*}

respectively. The corresponding multistate representation of this simplified process is given in Figure 1(b).

### 3.2. An AIPW estimator

The AIPW estimator (Robins *and others*, 1994) of the prediction error is defined here as


(3.9) \begin{align*} \label{aipw} \widehat{\rm PE}_{\text{AIPW}} (t_0)= \frac{1}{n} \sum_{i \in {\mathcal V}} \left[\frac{\Delta_i}{\hat\pi_i} \left|Y_i - \hat{Y}_i(\boldsymbol{X_i}; \hat{\boldsymbol{\theta}}) \right| + \left( 1- \frac{\Delta_i}{\hat\pi_i} \right) E \left\{ \left|Y_i - \hat{Y}_i(\boldsymbol{X_i}; \hat{\boldsymbol{\theta}}) \right| \bigm| \boldsymbol{X_i} \right\} \right]. \end{align*}

In typical applications, augmented inverse weighted estimators have a so-called “double-robustness” property which states that if the weight model or the event (prediction) model is correct then a consistent estimator for the parameter of interest is obtained. In the present setting, the weight is dependent on the event model and therefore if the event model is incorrect then the weight model must be incorrect. Therefore, in the present setting the double-robustness property is not present; see Section 7 for further comments. There is merit to investigating the empirical bias (EBIAS) and relative efficiency of the estimator in (3.9) however, and we do so in what follows. The AIPW method therefore yields a consistent estimator of the prediction error under the condition that only the event model is correctly specified.

## 4. ROC curves and the area under the curve

The ROC curve is widely used in health-related studies to examine the predictive performance of the classification based on patients’ status. It plots the true positive rate (TPR) and false positive rate (FPR) corresponding to a set of binary predictors and may help one select the optimal predictor with a desired accuracy. Consider a set of binary predictors of survival status at a specific time $t_0$, $ \hat Y(\boldsymbol{X}; \hat{\boldsymbol{\theta}}) = I(\mathcal{F}(t_0 | \boldsymbol{X}; \hat{\boldsymbol{\theta}}) > c)$, where $c \in (0,1)$. The TPR and FPR are defined as


(4.10) $$\begin{array}{c} \text{TPR}(c)=P(\hat{Y}=1|Y=1)=\frac{P(F(t_{0}|X;\hat{\theta })>c,T>t_{0})}{P(T>t_{0})},\; \end{array}$$

(4.11) $$\begin{array}{c} \text{FPR}(c)=P(\hat{Y}=1|Y=0)=\frac{P(F(t_{0}|X;\hat{\theta })>c,T⩽t_{0})}{P(T⩽t_{0})}, \end{array}$$

and the ROC curve is obtained by plotting $\text{TPR}(c)$ against $\text{FPR}(c)$ with the value of $c$ changing from 0 to 1. We can estimate the TPR and FPR in (4.10) and (4.11) by using inverse weighting methods to estimate the component probabilities. For example, the IPW and AIPW estimators of $P({\mathcal F}(t_0 | \boldsymbol{X}; \hat{\boldsymbol{\theta}}) > c, T > t_0)$ in the numerator of the TPR in (4.10) are


$$\begin{align*}
\frac{1}{n} \sum_{i \in {\mathcal V}} \frac{\Delta_i}{\hat\pi_i}I({\mathcal F}(t_0|\boldsymbol{X_i}; \hat \theta)>c, T_i>t_0)
\end{align*}$$

and


$$\begin{align*}
\frac{1}{n} \sum_{i \in {\mathcal V}} \left[ \frac{\Delta_i}{\hat\pi_i} I({\mathcal F}(t_0|\boldsymbol{X_i}; \hat \theta)>c, T_i > t_0) + \left( 1- \frac{\Delta_i}{\hat\pi_i}\right) E\left\{I({\mathcal F}(t_0|\boldsymbol{X_i}; \hat \theta)>c, T_i > t_0) \bigm| \boldsymbol{X_i}\right\} \right],
\end{align*}$$

respectively.

The area under the curve (AUC) is a summary measure of the ROC curve taking values from 0 to 1, with 0.5 representing a noninformative classification procedure (Hanley and McNeil, 1982); the closer the AUC is to 1 the greater the discriminative power of the predictive score. An alternative probabilistic interpretation of the AUC is apparent from the definition


(4.12) \begin{align*} \text{AUC} = P({\mathcal F}(\boldsymbol{X_i}; \boldsymbol\theta) > {\mathcal F}(\boldsymbol{X_j}; \boldsymbol\theta) \mid T_i > t_0, T_j \leqslant t_0), \end{align*}

which is the probability that a randomly selected individual $i$ who is event-free (i.e. for whom $T_i > t_0$) has a higher survival probability than a randomly selected individual $j$ who has experienced the event (i.e. for whom $T_j \leqslant t_0$) (Heagerty and Zheng, 2005; Uno *and others*, 2011).

As in the case of right-censored data, the ROC curve and the AUC statistic can be estimated by imputation, inverse probability weights, or augmented IPW methods. The estimators of the AUC can be written in a general form of


(4.13) \begin{align*} \begin{aligned} \hat{\text{AUC}} & = \frac{\displaystyle{ \sum\limits_{i \in {\mathcal V}} \sum\limits_{j \in {\mathcal V}} \biggl\{ I\Bigl({\mathcal F}(\boldsymbol{X_i}; \boldsymbol\theta) > {\mathcal F}(\boldsymbol{X_j}; \boldsymbol\theta) \Bigr) + 0.5 I(\boldsymbol{X_i} = \boldsymbol{X_j}) \biggr\} \hat\Psi(i)\hat\Phi(j) } }{ \displaystyle{ \sum\limits_{i \in {\mathcal V}} \sum\limits_{j \in {\mathcal V}} \hat\Psi(i) \hat\Phi(j)}}, \end{aligned} \label{auc} \end{align*}

where $\hat\Psi(i)$ is the estimator of $Y_i = I(T_i > t_0)$ and $\hat\Phi(j)$ is the estimator of $1- Y_j = I(T_j \leqslant t_0)$. An imputation-based estimator of the AUC is defined as in (4.13), but where


$$\begin{align*}\hat\Psi_\text{IMP}(i) =\Delta_i Y_i + \left( 1- \Delta_i \right) E\Bigl\{ Y_i \mid T_i \in [a_i^-, a_i^+], \boldsymbol{X}_i \Bigr\},\end{align*}$$

and


$$\begin{align*}\hat\Phi_\text{IMP}(j) = \Delta_j ( 1- Y_j) + \left( 1 - \Delta_j \right) E\Bigl\{ 1-Y_j \mid T_j \in [a_j^-, a_j^+], \boldsymbol{X}_j \Bigr\}\end{align*}$$

are used for $Y_i$ and $1-Y_j$, respectively.

The modeling scheme for the construction of the weights is as discussed earlier. The IPW estimator of the AUC corresponds to the use of


$$\begin{align*}\hat\Psi_\text{IPW}(i) =\frac{\Delta_i}{\hat\pi_i} Y_i
\quad \text{and} \quad
 \hat\Phi_\text{IPW}(j) = \frac{\Delta_j}{\hat\pi_j} (1- Y_j),\end{align*}$$

as estimators of $Y_i$ and $1-Y_j$, respectively, whereas the AIPW estimator of the AUC uses


$$\begin{align*}
\hat\Psi_\text{AIPW}(i)\,{=}\,\frac{\Delta_i}{\hat\pi_i} Y_i + \left(\!\! 1- \frac{\Delta_i }{\hat\pi_i} \!\right) E\Bigl(\! Y_i \mid \boldsymbol{X_i} \Bigr)
\quad \text{and} \quad
\hat\Phi_\text{AIPW}(j) = \frac{\Delta_j}{\hat\pi_j} ( 1- Y_j) + \left(\!\! 1- \frac{\Delta_j}{\hat\pi_j} \!\right) E\Bigl(\! 1-Y_j \mid \boldsymbol{X_j} \Bigr).\end{align*}$$

## 5. Simulation studies

### 5.1. Design and results of simulation studies for a Poisson assessment process

We next investigate the properties of these estimators of the performance of a prediction model based on case $K$ interval-censored data in a validation sample. Both the training and validation samples are generated using the identical set-up. Here, we consider the setting where the response is a failure time and there are three covariates denoted $X_{i1}$, $X_{i2}$, and $X_{i3}$. In Scenario I, we take them to have marginal standard normal distributions with $X_{i1} \perp X_{i2}$, $X_{i1} \perp X_{i3}$, and ${\rm corr}(X_{i2}, X_{i3})=0$ or 0.5. In Scenario II, the covariates are binary with $P(X_{ij}=1) = 0.5$, $j = 1, 2, 3$. In both scenarios given $(X_{i1}, X_{i2}),$ the event time $T_i$ follows a Weibull distribution with a proportional hazards formulation so


$$\begin{align*}
{\mathcal F}(t |X_{i1}, X_{i2}; \theta) =\exp\left\{ -(\lambda t)^\kappa \exp\left(X_{i1} \beta_1 + X_{i2} \beta_2 \right)
\right\},
\end{align*}$$

where $\theta = (\lambda, \kappa, \beta_1, \beta_2)'$; we set $\beta_1 = \log2$, $\beta_2= \log1.5,$ and $\kappa = 1.25$ and determine the value of $\lambda$ so that $P(T > 1) = {\mathcal F}(t) = E \{ {\mathcal F}(t |X_{i1}, X_{i2}; \theta)\} = 0.5$. We let $B$ denote the end of an administrative observation period and choose $B$ such that ${\mathcal F}(B) = 0.9$. The assessment process is taken to be a time-homogeneous Poisson process with rate


$$\begin{align*}
\lambda^a(s | X_{i1}, X_{i3}; \gamma) = \exp(\gamma_0 + X_{i1} \gamma_1 + X_{i3} \gamma_2 ),
\end{align*}$$

where $\gamma_1 =\log(1.1)$ and $\gamma_2 = \log(1.5)$ in Scenario I for the normal covariates and $\gamma_1 =\log(2)$ and $\gamma_2 = \log(2.5)$ in Scenario II for the binary covariates and $\gamma_0$ is set to ensure that the average number of assessments over (0, $B$) is controlled at $\mu = E\{ \int_0^B \lambda^a(s | X_{i1}, X_{i3}; \gamma) \mathrm d s \} = 10$.

The weights are estimated by modeling the event and assessment processes using (3.7) and (3.8). A pair of training and validation data sets each with a sample size of $m = 500$ are generated accordingly for both Scenarios I and II and we generate 100 replicates ($nsim = 100$). For each simulated training data set, a Weibull regression model was fitted for the event time under the assumption of a conditionally independent visit process (Cook and Lawless, 2018). Parametric and semiparametric models were fitted to the assessment process using a time-homogeneous and a semiparametric Andersen–Gill (AG) Poisson regression model (Andersen and Gill, 1982) respectively to the validation sample data. The unweighted and imputation-based estimators only depend on the model for the event process, while the IPW and AIPW estimators depend on both the event and observation processes. The corresponding estimators are denoted as IPW-TH, AIPW-TH when the time-homogeneous Poisson regression model is used for the visit process, and IPW-AG, AIPW-AG when the semiparametric AG model is used.

The EBIAS and empirical standard error (ESE) of the estimators of the prediction error at time $t_0$ are summarized in Table 1 as the first column of results ($\eta =1$), where $t_0$ value is taken to be the median of the marginal distribution of $T$. The biases of the unweighted estimators are large as expected with the imputation-based estimators performing well since the correct response model is fitted. Under correct specification of the assessment model, the proposed IPW and AIPW estimators have relatively small biases compared to the unweighted estimators, while the variability, as reflected by the ESE, is greater than that found for the imputation-based estimator. Note that the AIPW estimators are more efficient than the IPW estimators here, but this estimator relies on correct specification of the prediction model.

**Table 1.** EBIAS and standard error of estimators of prediction error; sample size $m = {\it 500}$, number of simulations $nsim = {\it 100}$. The gap times between two consecutive assessments are generated by a Gamma distribution with shape $\eta =1$ (Poisson Process), 1.25, and 2 (Modulated Renewal Process).

		Visit process					
		Poisson		Modulated renewal			
		$\eta = 1$		$\eta = 1.25$		$\eta = 2$	
CORR($X_{i2}$,$X_{i3}$)	METHOD	EBIAS	ESE	EBIAS	ESE	EBIAS	ESE
		Scenario I: Normal covariates					
0	Unweighted	−0.0425	0.0246	−0.0409	0.0216	−0.0385	0.0209
	Imputation	0.0006	0.0190	0.0008	0.0172	0.0004	0.0168
	IPW-TH	0.0039	0.0286	0.0143	0.0264	0.0333	0.0267
	IPW-AG	0.0043	0.0282	0.0135	0.0263	0.0304	0.0265
	AIPW-TH	0.0023	0.0251	0.0044	0.0233	0.0046	0.0237
	AIPW-AG	0.0027	0.0251	0.0046	0.0232	0.0043	0.0236
0.5	Unweighted	−0.0448	0.0260	−0.0483	0.0200	−0.0428	0.0239
	Imputation	−0.0002	0.0202	−0.0033	0.0168	−0.0018	0.0202
	IPW-TH	0.0030	0.0297	0.0074	0.0229	0.0329	0.0292
	IPW-AG	0.0032	0.0296	0.0069	0.0228	0.0302	0.0289
	AIPW-TH	0.0020	0.0260	−0.0030	0.0217	0.0042	0.0261
	AIPW-AG	0.0024	0.0259	−0.0027	0.0217	0.0040	0.0259
		Scenario II: Binary covariates					
0	Unweighted	−0.0358	0.0238	−0.0282	0.0246	−0.0306	0.0274
	Imputation	−0.0035	0.0201	0.0031	0.0196	0.0006	0.0224
	IPW-TH	−0.0014	0.0269	0.0190	0.0303	0.0356	0.0318
	IPW-AG	−0.0013	0.0266	0.0177	0.0297	0.0324	0.0315
	AIPW-TH	−0.0033	0.0222	0.0047	0.0244	0.0007	0.0277
	AIPW-AG	−0.0016	0.0222	0.0063	0.0242	0.0025	0.0276
0.5	Unweighted	−0.0340	0.0250	−0.0358	0.0250	−0.0274	0.0225
	Imputation	−0.0008	0.0201	−0.0039	0.0211	0.0017	0.0184
	IPW-TH	−0.0012	0.0269	0.0072	0.0289	0.0424	0.0300
	IPW-AG	−0.0010	0.0267	0.0069	0.0291	0.0390	0.0288
	AIPW-TH	−0.0021	0.0246	−0.0056	0.0233	0.0062	0.0237
	AIPW-AG	−0.0004	0.0245	−0.0036	0.0233	0.0080	0.0233

In Table 2, we report the empirical properties of the estimators of the AUC with a similar layout of the results. The column of results for the Poisson visit process ($\eta = 1$) shows a similar pattern of behavior as in the analogous column of Table 1 with one exception; the ESE of the estimates based on AIPW (either under the time-homogeneous or semiparametric visits process model) are not smaller than the estimators based on the IPW approach.

**Table 2.** EBIAS and standard error of estimators of the area under the ROC; sample size $m = {\it 500}$, number of simulations $nsim = {\it 100}$. The gap times between two consecutive assessments are generated by a Gamma distribution with shape $\eta ={\it 1}$ (Poisson Process), 1.25, and 2 (Modulated Renewal Process).

		Visit process					
		Poisson		Modulated renewal			
		$\eta = 1$		$\eta = 1.25$		$\eta = 2$	
CORR($X_{i2}$,$X_{i3}$)	METHOD	EBIAS	ESE	EBIAS	ESE	EBIAS	ESE
		Scenario I: Normal covariates					
0	Unweighted	0.0466	0.0216	0.0437	0.0222	0.0414	0.0227
	Imputation	0.0001	0.0184	−0.0017	0.0185	−0.0026	0.0194
	IPW-TH	−0.0018	0.0235	−0.0056	0.0240	−0.0068	0.0250
	IPW-AG	−0.0023	0.0235	−0.0060	0.0240	−0.0065	0.0249
	AIPW-TH	−0.0021	0.0246	−0.0056	0.0249	−0.0060	0.0271
	AIPW-AG	−0.0025	0.0246	−0.0059	0.0249	−0.0057	0.0269
0.5	Unweighted	0.0507	0.0242	0.0552	0.0201	0.0474	0.0229
	Imputation	0.0001	0.0216	0.0043	0.0191	−0.0005	0.0205
	IPW-TH	−0.0014	0.0254	0.0035	0.0226	−0.0062	0.0252
	IPW-AG	−0.0021	0.0254	0.0031	0.0227	−0.0061	0.0252
	AIPW-TH	−0.0012	0.0252	0.0045	0.0241	−0.0056	0.0271
	AIPW-AG	−0.0017	0.0251	0.0042	0.0242	−0.0054	0.0269
		Scenario II: Binary covariates					
0	Unweighted	0.0406	0.0260	0.0331	0.0279	0.0351	0.0289
	Imputation	0.0016	0.0218	−0.0044	0.0233	−0.0019	0.0229
	IPW-TH	0.0016	0.0251	−0.0070	0.0276	−0.0035	0.0276
	IPW-AG	−0.0002	0.0251	−0.0087	0.0275	−0.0052	0.0276
	AIPW-TH	0.0012	0.0245	−0.0072	0.0279	−0.0034	0.0291
	AIPW-AG	−0.0006	0.0244	−0.0089	0.0276	−0.0052	0.0288
0.5	Unweighted	0.0436	0.0264	0.0470	0.0257	0.0315	0.0245
	Imputation	0.0004	0.0224	0.0044	0.0226	−0.0056	0.0206
	IPW-TH	0.0017	0.0268	0.0065	0.0253	−0.0107	0.0248
	IPW-AG	−0.0008	0.0268	0.0037	0.0254	−0.0133	0.0245
	AIPW-TH	0.0015	0.0270	0.0064	0.0257	−0.0119	0.0268
	AIPW-AG	−0.0009	0.0268	0.0037	0.0256	−0.0143	0.0262

### 5.2. Design and results of studies for renewal assessment processes

We next investigate the impact of misspecification of the assessment model. We consider the scenario in which the assessment process is governed by a non-Markov renewal process but analysis of the assessment process is still based on a parametric or semiparametric Poisson process. The event times are generated as described in Section 5.1, but here the gap times between consecutive assessments are Gamma distributed with shape $\eta$ and rate $\exp(\gamma_0 + X_{i1} \gamma_1 + X_{i3} \gamma_2 )$, where $\eta = 1.25$ and 2; the values of the other parameters are the same as in Section 5.1 for both the normal and binary covariates. For each parameter setting, 100 data sets with sample sizes of $m = 500$ are simulated. The empirical performance of the estimators of the prediction error under this setting are summarized in the second two sets of columns of Table 1 ($\eta = 1.25$ and 2). Since the assessment process is non-Markov, the further the shape parameter $\eta$ is from 1, the greater the difference from the time-homogeneous Poisson process, and hence the greater the extent of misspecification. We find that EBIASes of the IPW-TH and IPW-AG estimators increase as $\eta$ increases, but the AIPW-TH and AIPW-AG estimators maintain relatively small bias. The ESEs of the AIPW estimators are smaller than those of the IPW estimators, which again demonstrates the improved efficiency of the AIPW estimators for the prediction error. The EBIAS and standard error (ESE) of the unweighted, IMPUTED, IPW, and AIPW estimators of the AUC at the landmark time $t_0$ are summarized in the last two sets of columns Table 2 for both scenarios where similar findings can be seen.

In broad terms, we found that when the joint model was misspecified there was a consequent EBIAS in the IPW estimators, but that this bias is smaller than that of the unweighted estimator for the misspecification considered here. When the event model is correctly specified, but the assessment model is misspecified, the AIPW estimators have a comparable performance with those under correct specification of the assessment model; the bias remains small and the standard errors are modest.

## 6. Prediction of arthritis mutilans in psoriatic arthritis

The University of Toronto Psoriatic Arthritis Clinic registry was launched in 1977 to study this complex disease (Gladman *and others*, 2008; Gladman and Chandran, 2011). Patients in this registry undergo a detailed clinical and radiological examination upon entry to the clinic, and provide serum samples for genetic testing. Follow-up clinical and radiological assessments are scheduled annually and biannually, respectively, in order to track changes in joint damage. At each radiological assessment the degree of damage is recorded in 64 joints on a five-point scale. To date, 1495 patients have been recruited to the registry, and 1185 of these have undergone genetic testing to determine their human leukocyte antigen (HLA) profile. The genotypes of HLA-A, HLA-B, and HLA-C alleles were collected, and a total of 70 HLA markers were identified as of interest a prior; 15 of these markers had a frequency in the sample of less than 1% and so were excluded from further consideration. While there is no clinical consensus on how precisely to define arthritis mutilans, it represents a state of significant joint damage arising from an extreme form of the disease; here, we define it as present if an individual has 5 or more joints with the advanced stage of damage according to the modified Steinbrocker score (Rahman *and others*, 1998).

**Fig. 3.**
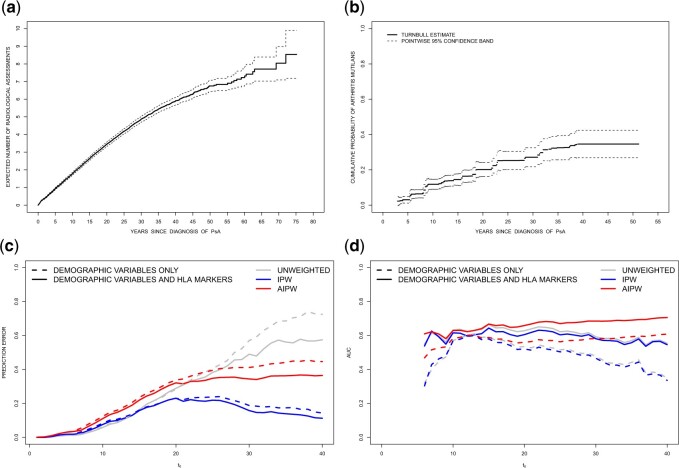
Panel (a) contains a plot of the expected number of radiological assessments since the time of diagnosis with psoriatic arthritis. Panel (b) contains a plot of the Turnbull estimate with a pointwise 95% confidence band for the marginal cumulative distribution function of the time from disease onset to arthritis mutilans. Panel (c) contains a plot of the estimates of the prediction error and panel (d) shows the area under the ROC versus $t_0$ with a binary predictor $I(P(T > t_0) > 0.5)$ for the models with (i) demographic variables only or (ii) demographic variables and HLA markers. Training data including randomly selected 508 subjects is used to develop the prediction model and the test data containing the remaining 507 subjects is used to evaluate the predictive performance. The assessment model is fitted based on the entire data.

We consider a data set containing 1015 patients from the University of Toronto Psoriatic Arthritis Clinic with the median time from the diagnosis of psoriatic arthritis to last radiological assessment being 12.0 years (lower quartile $=5.1$, upper quartile $= 22.5$). Plot of the Nelson–Aalen estimate of the mean function for the number of radiological assessments is given in Figure 3(a). Among the 1015 patients, a total of 874 (86.1%) patients were not observed to develop arthritis mutilans and hence provided right-censored times, whereas 141 (13.9%) patients were known to develop arthritis mutilans yielding interval-censored times since they had a visit with a damaged joint count of 5 or greater. The 25th, 50th, and 75th percentiles of the censoring interval lengths for the arthritis mutilans patients were 2.6, 7.9, and 15.0 years respectively. Figure 3(b) contains a nonparametric estimate and pointwise 95% confidence bands (Turnbull, 1976) for the cumulative distribution function of the time from onset of psoriatic arthritis to arthritis mutilans. The estimate reflects a steadily increasing risk with roughly 20% of psoriatic arthritis patients developing the condition within 20 years of disease onset.

Our interests lie in identifying which among the 55 HLA markers are associated with the development of arthritis mutilans from the onset of psoriatic arthritis, and assessing the predictive performance of these models.

We partition the total of 1015 patients into training ($n_0 = 508$) and validation ($n = 507$) samples randomly. We adopt a proportional hazards model with a Weibull baseline hazard to develop the prediction model of the time from onset of psoriatic arthritis to arthritis mutilans by using the training sample. All models controlled for four demographic variables including age at clinic entry, sex, family history of psoriasis, and family history of psoriatic arthritis. Semiparametric analysis is carried out to model the inspection process, that is, modeling the gap times between two consecutive inspection times by AG model. The covariates in the inspection process model are the four demographic variables and the set of the 19 HLA markers.

Having fitted these models, we next apply the inverse weighting approach to estimate the prediction errors and to assess the discriminative abilities for the models in the validation sample. Figure 3(c) shows the results in terms of prediction error curves by using the unweighted, IPW, and AIPW with $t_0$ ranging from 0 to 40 years after the diagnosis of psoriatic arthritis. The unweighted estimates are greater than the weighted estimates since the unweighted estimators do not account for the unclassified portion in the sample. Figure 3(d) shows the estimates of the area under the ROC as a function of $t_0$. The ROC curves at $t_0 =10$ and 20 years after diagnosis of psoriatic arthritis for both models with IPW and AIPW estimators are shown in Figure S1 of Supplementary material available at *Biostatistics* online; the corresponding estimates of the AUC’s are also given in the legend. We conclude from these figures that the model including HLA markers has a better predictive performance in terms of a higher AUC and lower prediction error compared with the model including the demographic variables only.

## 7. Discussion and future research

In this article, we described imputation techniques and use of inverse probability weighting to estimate predictive performance in settings when validation samples have case $K$ interval-censored data arising from intermittent assessments of individuals. The simulation studies demonstrated that the proposed imputation-based, IPW, and AIPW estimators led to better performance compared to the simple unweighted estimators. For many data sets involving interval censoring only the left and right endpoints of the censoring intervals are reported; such information are insufficient to model the inspection process and implement the proposed method. Implementation hinges critically on the availability of all inspection times over the course of observation (i.e., case $K$ interval censoring). In the motivating study such data are available, and the proposed methods were illustrated by an application in which a semiparametric analysis is carried out to model the inspection process and a parametric proportional hazards model was used to model the event time with a Weibull baseline hazard function. More flexible approaches could be considered for model fitting including the use of models with piecewise constant baseline hazards or penalized regression to select the prediction model as in Wu and Cook (2015).

The expressions for the estimated weights in Sections 3.1 and 3.2 are based on the joint model for the response and observation processes, whereas the imputation-based estimator estimates the conditional probability of failure by the landmark time $t_0$ given the respective censoring interval. A hybrid approach has been suggested by a referee for the setting in which the loss to follow-up time is reported. Here, attention could be restricted to individuals who have not been lost to follow-up before the landmark time. An imputation-based approach could then be adopted among these individuals with inverse probability of censoring weights (Graf *and others*, 1999; Hothorn *and others*, 2006; Gerds and Schumacher, 2006; Lawless and Yuan, 2010) used to address the fact that this imputation-based estimator is applied to a biased sub-sample of individuals. A study of the robustness and efficiency of this approach compared to the methods presented here would be of interest.

Wu and Cook (2018) develop methods for variable selection with truncated and interval-censored data. While we have dealt with the latter complication here, it is less clear how one might assess predictive accuracy when samples are chosen subject to truncation, but this feature is often present in the development of predictive models based on data acquired from different sources with distinct selection criteria. This is a topic of ongoing research.

The availability of external validation data is highly desirable when we consider the assessment of the predictive performance. There are several clinical registries of individuals with psoriatic arthritis in Spain (Queiro *and others*, 2003), Ireland (Winchester *and others*, 2012), and Newfoundland (Rahman *and others*, 2011), most of which are devoted to some form of genetic research aiming to identify prognostic markers. We may consider use of such external validation data sets and are currently investigating the extent of follow-up in these registries. An issue with the Newfoundland cohort is that the distribution of genetic markers and other attributes among the members of this registry is different than those individuals in the University of Toronto Psoriatic Arthritis Clinic Registry. As a result, we might expect the estimated prediction error based on such an external validation sample to be quite different than the estimates obtained by cross-validation based on the Toronto registry. Approaches for calibrating the covariate distribution for a more reasonable expectation of the predictive accuracy in this setting are also of interest but these will require detailed information of the Newfoundland cohort.

## 8. Software

Software in the form of R code, together with a sample input data set is available on GitHub and can be requested from the corresponding author (ywu@nankai.edu.cn).

## Supplementary Material

kxaa011_Supplementary_Data
