# Supplementary material for: Assessing the accuracy of predictive models with interval-censored data
Source: Biostatistics. 2020 Mar 14;23(1):18–33. doi: 10.1093/biostatistics/kxaa011 (PMC8974097; doi:10.1093/biostatistics/kxaa011)
Supplement: kxaa011_Supplementary_Data [file kxaa011_supplementary_data.zip › biosts-19102-File001.pdf]

Biostatistics (2019), **0**, 0, *pp.* 1–5
doi:10.1093/biostatistics/supplementary

Supplementary Material for Assessing the Accuracy of Predictive Models with Interval-Censored Data

YING WU*

School of Statistics and Data Science, Nankai University, Tianjin, China

ywu@nankai.edu.cn

RICHARD J. COOK

Department of Statistics and Actuarial Science,

University of Waterloo, Waterloo, Ontario, N2L 3G1, Canada

APPENDIX

Appendix A:. Development of A Prediction Model With Training Data

In this section, we consider the development of a prediction model based on a training data, which can be made up of case I, II or K interval-censored data (Sun, 2006). We consider case K interval-censored failure time data here which arises from longitudinal studies involving intermittent observation. We describe the construction of the full likelihood and outline the assumptions necessary to justify the partial likelihood typically used when fitting regression models for case K interval-censored data.

Let $\boldsymbol{X}$ be a $p \times 1$ covariate vector used in the construction of a risk score for prediction regarding an event time T. An event time model can be represented by a progressive 2-state stochastic process with states labeled 0 and 1 representing the conditions of being event-free and

© The Author 2019. Published by Oxford University Press. All rights reserved. For permissions, please e-mail: journals.permissions@oup.com

post-event respectively. If T is the random event time we let $Z(s) = I(T \leqslant s)$ record the state occupied at time s and $\{Z(s), 0 \leqslant s\}$ be the corresponding stochastic process; the history of the 2-state process is denoted by $\mathcal{Z}(t) = \{Z(s), 0 \leqslant s \leqslant t\}$.

Suppose the intention is to follow an individual up to an administrative censoring time B, and let R ($R < B$) denote a random loss to follow-up time. Then if $C = \min(R, B)$, we define $C(s) = I(C \leqslant s)$ and let $\{C(s), 0 < s\}$ denote the counting process for the censoring time. Individuals are observed intermittently at clinic visit times denoted by $0 = a_0 < a_1 < \cdots$ where A_r denotes the random time of the rth visit and a_r is its realized value, $r = 1, \ldots$. We let $A(s) = \sum_{r=1}^{\infty} I(a_r \leqslant s)$ be a right-continuous process counting the number of post-baseline assessments over $[0, s]$ and let $dA(s) = A(s) - A(s^-) = 1$ if an assessment is made at time s and $dA(s) = 0$ otherwise. Since visits can only be made for individuals still on study, the visit process terminates at C so we observe $d\bar{A}(s) = Y(s)dA(s)$, where $Y(s) = I(s \leqslant C)$ and $\bar{A}(t) = \int_0^t d\bar{A}(s)$. When considered jointly we refer to $\{C(s), A(s), 0 < s\}$ as the *observation process* where we observe $Z(s)$ only if $d\bar{A}(s) = 1$.

We consider here a joint model for the two-state event and observation processes and let $\mathcal{H}(t) = \{C(u), A(u), Z(u), 0 \leqslant u \leqslant t, X\}$ denote the *complete history* at time t. Note $\mathcal{H}(t)$ is not observable since $Z(s)$ is only known at the assessment times. If $0 \leqslant a_0 < a_1 < \cdots < a_{A(s)}$ denote the realized assessment times over $[0, s]$, then the *observed history* is $\mathcal{H}^\circ(t) = \{C(u), \bar{A}(u), 0 \leqslant u \leqslant t; (a_j, Z(a_j)), j = 1, \ldots, \bar{A}(t), X\}$ and we let $\mathcal{Z}^\circ(t) = \{(a_j, Z(a_j)), j = 0, 1, \ldots, \bar{A}(t), X\}$.

We assume that loss to follow-up (i.e. right-censoring) is conditionally independent and non-informative (Kalbfleisch and Prentice, 2011) with intensity

$$\lim_{\Delta t \downarrow 0} \frac{P(\Delta C(t) = 1 \mid \mathcal{H}(t^-))}{\Delta t} = I(t \leqslant C)\, \lambda^c(t \mid \mathcal{H}^\circ(t^-)) \; , \tag{A.1}$$

where $\Delta C(t) = C(t + \Delta t^-) - C(t^-)$. Cook and Lawless (2018) define a *conditionally independent*

visit process (CIVP) in this context as one for which

$$\lim_{\Delta t \downarrow 0} \frac{P(\Delta \bar{A}(t) = 1 \mid \mathcal{H}(t^-))}{\Delta t} = I(t < C)\, \lambda^a(t \mid \mathcal{H}^\circ(t^-)) \;, \tag{A.2}$$

where $\Delta \bar{A}(t) = \bar{A}(t + \Delta t^-) - \bar{A}(t^-)$. Note that the visit process intensity can depend on the number and times of previous visits as well as the states occupied at these visits.

The likelihood for an individual with m follow-up visits realized at $a_1 < \cdots < a_m \leqslant C$ is

$$L \propto \cdot L_C \cdot L_A \cdot \prod_{j=1}^{m} P(Z(a_j) \mid a_j, d\bar{A}(a_j) = 1, \mathcal{H}^\circ(a_j^-)) \;, \tag{A.3}$$

where

$$L_C \propto \lambda^c(C|\mathcal{H}^\circ(c)) \exp\Big(- \int_0^\infty Y(u) \lambda^c(u|\mathcal{H}^\circ(u^-))\, du \Big) \tag{A.4}$$

and

$$L_A \propto \prod_{j=1}^{m} \Big[\lambda^a(a_j \mid \mathcal{H}^\circ(a_j^-)) \Big] \exp\Big(- \int_{a_0}^\infty Y(u) \lambda^a(u \mid \mathcal{H}^\circ(u^-))\, du \Big) \,. \tag{A.5}$$

If the assessment and loss to follow-up processes are non-informative we base analysis on the partial likelihood

$$PL \propto \prod_{j=1}^{m} P(Z(a_j) \mid a_j, d\bar{A}(a_j) = 1, \mathcal{H}^\circ(a_j^-)) \;. \tag{A.6}$$

To write the contributions to (A.6) in terms of the event time model of interest we require the conditional probabilities $P(Z(a_j)|a_j, d\bar{A}(a_j) = 1, \mathcal{H}^\circ(a_j^-))$ satisfy

$$P(Z(a_j) \mid a_j, d\bar{A}(a_j) = 1, \mathcal{H}^\circ(a_j^-)) = P(Z(a_j) \mid a_j, \mathcal{Z}^\circ(a_j^-)) \,, \tag{A.7}$$

where on the right-hand side the probability is computed as if the visit times a_j, $j = 0, 1, 2, \ldots$ were fixed in advance. This means that the intensities governing the event time process are the same whether the process is under the particular observation scheme or not (Cook and Lawless, 2019). If (A.7) is satisfied the partial likelihood based on the multistate model is

$$\prod_{j=1}^{m} P(Z(a_j) \mid a_j, \mathcal{Z}^\circ(a_j^-)) \,. \tag{A.8}$$

We consider the goal of using fixed covariates to model event occurrence and adopt a simple hazard-based model for T with

$$\lim_{\Delta t \downarrow 0} \frac{P(Z(t+\Delta t^-)=1|Z(t^-)=0,\boldsymbol{X})}{\Delta t} = I(t \leqslant T)\, h(t|\boldsymbol{X}) \ ,$$

which we parameterize with θ. Note that for the purpose of computing (A.8) for a progressive $0-1$ we can reduce the number of contributions. If $0 = a_0 < a_1 < \cdots < a_m$ are the m assessment times over $[0, C]$, let $L = \max_{Z(a_j)=0} a_j$ so that the censoring interval for T is $[L, R]$, where R is the assessment after L if $L < a_m$, and otherwise we set $R = \infty$. Since the response model is a progressive $0-1$ process, the observed data necessary to compute the partial likelihood (A.8) can be written simply as $D = \{L, R, \boldsymbol{X}\}$. An individual's contribution to (A.8) then reduces to

$$PL \propto [\mathcal{F}(L \mid \boldsymbol{X}) - \mathcal{F}(R \mid \boldsymbol{X})] \ ,$$

where $\mathcal{F}(t|\boldsymbol{X}) = \exp(-H(t|\boldsymbol{X}))$ is the survivor function with $H(t|\boldsymbol{X}) = \int_0^t h(s|\boldsymbol{X})ds$.

With a set $\mathcal{T}$ of n_0 independent individuals in a training sample let $D_{\mathcal{T}} = \{(L_i, R_i, \boldsymbol{X_i}), i \in \mathcal{T}\}$ denote the training data used for the development of a prediction model. The partial likelihood then reduces to

$$PL(\theta) \propto \prod_{i \in \tau} [\mathcal{F}(a_i^- \mid \boldsymbol{X_i};\boldsymbol{\theta}) - \mathcal{F}(a_i^+ \mid \boldsymbol{X_i};\boldsymbol{\theta})] \qquad \text{(A.9)}$$

and $\widehat{\boldsymbol{\theta}}$ denotes the maximum likelihood estimator.

Appendix B:. Modeling the Observation Process

In this section, we consider the model fitting for the observation process using the validation data, which is also case K interval-censored data. Let $D_{\mathcal{V}} = \{(a_{ij}, Z(a_{ij}), j = 1, \ldots, m_i, \boldsymbol{X_i}, C_i, \delta_i), i \in \mathcal{V}\}$ denote the validation data, where $\delta_i = I(R_i < B_i)$ is an indicator of random loss to follow-up (censoring).

The predictions are based on the risk scores or the fitted prediction model as described in Appendix A using the training data.

The weights used to construct the IPW and AIPW estimator are developed by joint consideration of the event and observation processes; see Section 3. The weight can be written as

$$P(\Delta_i = 1 | T_i \leqslant t_0, \boldsymbol{X_i}) = \int_0^{t_0} \left[\int_t^{t_0} \lambda^a(u \mid \mathcal{H}^\circ(u^-)) \exp\left\{ - \int_t^u \lambda^a(v \mid \mathcal{H}^\circ(v^-)) dv \right.\right.$$
$$\left.\left. - \int_0^u \lambda^c(v \mid \mathcal{H}^\circ(v^-) dv) \right\} du \right] \times f(t | T_i \leqslant t_0, \boldsymbol{X_i}) dt \ .$$
$$P(\Delta_i = 1 | T_i > t_0, \boldsymbol{X_i}) = \int_{t_0}^{\infty} \lambda^a(u \mid \mathcal{H}^\circ(u^-)) \exp\left\{ - \int_{t_0}^u \left[\lambda^a(v \mid \mathcal{H}^\circ(v^-)) + h(v | \boldsymbol{X_i}) \right] \, \mathrm{d}v \right.$$
$$\left. - \int_0^u \lambda^c(v \mid \mathcal{H}^\circ(v^-)) \, \mathrm{d}v \right\} \, \mathrm{d}u \ .$$

To estimate the weights used to construct the IPW and AIPW estimator, one must estimate the hazard of the event process $h(\cdot)$ and the intensities of the observation processes $\lambda^a(\cdot)$ and $\lambda^c(\cdot)$.

To estimate the intensities of the censoring and assessment processes, one can by maximizing the partial likelihoods in (A.4) and (A.5) using the validation data $D_{\mathcal{V}}$ to obtain the estimates $\widehat{\lambda}^a(\cdot)$ and $\widehat{\lambda}^c(\cdot)$.

[Fig. 1 about here.]

References

Cook, R. J. and Lawless, J. F. (2018). *Multistate Models for the Analysis of Life History Data*. Chapman and Hall/CRC.

Cook, R. J. and Lawless, J. F. (in press, 2019). Independence conditions and the analysis of life history studies under intermittent observation. *Biostatistic*.

Kalbfleisch, J. D. and Prentice, R. L. (2011). *The Statistical Analysis of Failure Time Data*, Volume 360. John Wiley & Sons.

Sun, J. (2006). *The Statistical Analysis of Interval-Censored Failure Time Data*. Springer, New York.

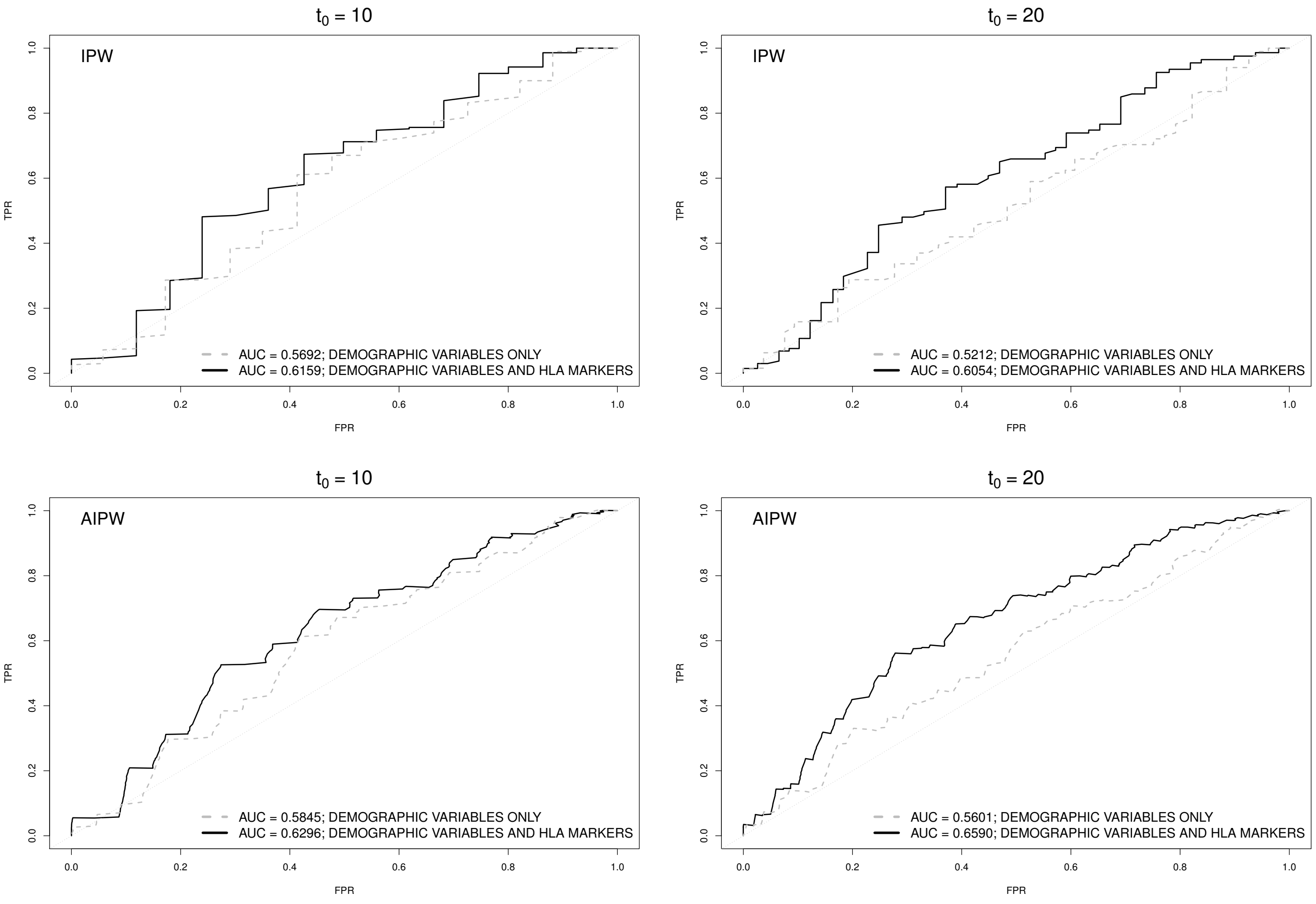

Fig. S1. ROC curves estimated at $t_0 = 10$ and 20 years under IPW and AIPW methods using prediction models with demographic variables only as well as both demographic variables and HLA markers.
